# Supplementary material for: Acrometastases to the Hand: A Systematic Review
Source: Medicina (Kaunas). 2021 Sep 9;57(9):950. doi: 10.3390/medicina57090950 (PMC8471162; doi:10.3390/medicina57090950)
Supplement: Supplementary file 1 [file medicina-57-00950-s001.zip › medicina-1271929-supplementary.pdf]

Authors	Year	No. Patients	Age/Sex	Metastatic location	Primary tumor	Interval time from primary tumor diagnosis to acrometastasis (months)	Metastases treatment	Survival (months)
Kosuda et al. [34]	1986	2	61/M	Left ring finger, distal phalanx	Esophageal squamous cell carcinoma	not specified	none	not reported
			47/F	Left middle finger, distal phalanx	Bronchogenic squamous cell carcinoma	not specified	none	not reported
Vijayakumar and Creditor [35]	1986	1	91/F	Right thumb distal phalanx	Breast carcinoma	12	Radiotherapy	3
Buckley and Brown [36]	1987	2	78/F	Left trapezium and trapezoid	Sigmoid colon adenocarcinoma	48	Chemotherapy	not reported
			61/F	Left little finger, proximal phalanx	Transverse colon adenocarcinoma	24	Radiotherapy, amputation	2
Henderson [37]	1987	1	63/M	Left thumb, distal phalanx	Bronchogenic squamous cell carcinoma	none	Amputation of distal phalanx	not reported
Hindley and Metcalfe [38]	1987	1	44/M	Right little finger, middle phalanx	Colon adenocarcinoma	24	Ray amputation	not reported
Bourne et al. [39]	1988	1	74/F	Right second, third, and fourth metacarpals	Colon adenocarcinoma	not specified	Radiotherapy	2
Craigen and Chesney [40]	1988	1	37/M	Right hamate	Gastric adenocarcinoma	36	not reported	not reported
Dalicho et al. [41]	1988	1	64/F	Right little finger, distal phalanx	Uterine cervices squamous cell carcinoma	0.7	Amputation of distal phalanx	1
Haas et al. [42]	1988	1	51/F	Right little finger, proximal phalanx	Esophageal squamous cell carcinoma	not specified	Radiotherapy	6
Müller et al. [43]	1988	1	77/M	Left hand, soft tissue	Rectal adenocarcinoma	24	Radiotherapy	not reported
Sanjay et al. [44]	1988	1	10/M	Left third metacarpal	Tibial small cell osteogenic sarcoma	not specified	none	not reported
Tenenbaum et al. [45]	1988	1	65/M	Right capitate and hamates	Esophageal squamous cell carcinoma	not specified	Radiotherapy	not reported
Sim [46]	1989	1	49/F	Right wrist	Bronchogenic squamous cell carcinoma	none	Amputation	7
Farouk et al. [47]	1990	1	66/M	Right ring finger, distal phalanx	Bronchogenic squamous cell carcinoma	none	PIP joint disarticulation	not reported
Heymans et al. [48]	1990	1	65/M	Right hand, dorsal soft tissue	Vesicourethral junction transitional cell carcinoma	none	Wide excision and skin graft	4
Lederer et al. [49]	1990	1	51/M	Right trapezium	Bronchogenic carcinoma	3	Excision of trapezium and trapezoid	not reported
Letanche et al. [50]	1990	2	53/M	Fifth metacarpal	Bronchial large cell carcinoma	not specified	Chemotherapy	2
			59/M	Right middle finger, middle phalanx	Bronchogenic squamous cell carcinoma	not specified	Amputation	11

Stone and Davies [26]	1990	1	51/F	Left little finger, distal and middle phalanges	Bronchogenic squamous cell carcinoma	not specified	Finger amputation	6
Turan et al. [51]	1990	1	43/F	Right little finger, proximal, middle and distal phalanges	Ovarian endometrioid carcinoma	none	MCP joint disarticulation	1.6
Desmanet et al. [52]	1991	2	48/M	Right middle finger, distal phalanx	Esophageal cancer	12	Amputation	2
			62/M	Right hamate	Bronchogenic cancer	not specified	Radiotherapy	10
Henkert and Berge [53]	1991	2	53/M	Left little finger, distal phalanx	Sigmoid colon adenocarcinoma	not specified	Amputation	12
			52/M	Right little finger, proximal phalanx	Unknown origin	none	not reported	4
Kolomiets and Lytkin [54]	1991	1	50/M	Left index finger, proximal phalanx	Bronchogenic small cell carcinoma	none	not reported	not reported
Moutet et al. [55]	1991	1	56/M	Right capitate	Esophageal squamous cell adenocarcinoma	9	Excision of the capitate	24
Rochet et al. [56]	1991	1	62/M	Left thumb, distal phalanx	Bronchogenic adenocarcinoma	none	Amputation	not reported
Troncoso et al. [57]	1991	1	53/M	Left index finger, distal phalanx	Renal cell carcinoma	24	DIP joint disarticulation	7
Bloom et al. [58]	1992	1	51/F	Both hands (not specified)	Breast carcinoma	not specified	not reported	not reported
DiSpaltro et al. [59]	1992	1	41/M	Right little finger, pulp	Gastric adenocarcinoma	5	Amputation of distal phalanx	not reported
Hayes et al. [60]	1992	1	76/M	Right first metacarpal	Transitional cell carcinoma, right renal pelvis	none	Transmetacarpal amputation	1
Jebson et al. [61]	1992	1	77/M	Left middle finger, pulp	Renal cell adenocarcinoma	none	PIP joint disarticulation	4
Kobus et al. [62]	1992	1	65/F	Left third metacarpal	Renal cell carcinoma	24	Ray amputation	
Lambert et al. [63]	1992	1	36/M	Right and left ring fingers, subungual	Femur chondrosarcoma	24	Surgical excision	3
Rousseau et al. [64]	1992	1	72/M	Left little finger, soft tissue of the proximal phalanx	Bronchogenic adenocarcinoma	5	Excision	not reported
Bibi et al. [65]	1993	1	60/M	Right little finger, distal phalanx	Renal cell carcinoma	36	PIP joint disarticulation	not reported
Marya et al. [66]	1993	1	62/M	Left thumb, distal phalanx	Bladder transitional cell carcinoma	6	none	8
Moens et al. [67]	1993	1	65/M	Right little finger, distal phalanx	Bronchogenic squamous cell carcinoma	4	PIP joint disarticulation	5
Roncaglio and Arena [68]	1993	2	44/M	Left lunate	Bronchogenic carcinoma	none	not reported	not reported
			55/M	Right ring finger, distal phalanx	Bronchogenic squamous cell carcinoma	not specified	Amputation	26

Knapp and Abdul-Karim [69]	1994	2	89/F	Right index finger, middle phalanx	Bronchogenic adenocarcinoma	none	Radiotherapy	4
			38/M	Left ring finger, middle phalanx	Gastroesophageal junction	not specified	Radiotherapy, chemotherapy	3
Walsh et al. [70]	1994	1	46/M	Right index finger, distal phalanx	Bladder transitional cell carcinoma	24	Amputation through the proximal phalanx	not reported
Abrahams [3]	1995	3	62/M	Right thumb, proximal phalanx	Bronchogenic adenocarcinoma	none	Radiotherapy, chemotherapy	not reported
			63/M	Right fourth metacarpal	Renal cell carcinoma	none	not reported	not reported
			59/F	Left lunate	Bronchogenic squamous cell carcinoma	none	not reported	not reported
De Maeseneer et al. [71]	1995	1	60/M	Right and left thumbs, distal phalanges	Bronchogenic squamous cell carcinoma	3	not reported	not reported
Castello et al. [72]	1996	6	68/M	Right thumb, distal phalanx	Hard palate squamous cell carcinoma	none	Amputation through proximal phalanx	10
			65/M	Left ring finger, proximal phalanx, third metacarpal	Renal cell carcinoma	none	Chemotherapy	not reported
			67/M	Right wrist, ulceration of the ulnar side	Nasopharynx squamous cell carcinoma	15	Excision of the ulcer, skin graft	8
			65/M	Right index finger, distal phalanx	Laryngeal squamous cell carcinoma	11	Distal phalanx amputation	6
			74/M	Right thumb, distal phalanx	Bronchogenic squamous cell carcinoma	24	Amputation through proximal phalanx	3
			68/F	Right middle finger, distal phalanx	Bronchogenic caricnoma	48	Amputation	7
Hetzel et al. [73]	1996	1	31/F	Left little finger, distal phalanx	Uterine choriocarcinoma	none	Chemotherapy	not reported
Königsberger and Goth [74]	1996	1	54/M	Right hamate	Bronchogenic small cell carcinoma	none	Chemotherapy	16
Saglike et al. [75]	1996	1	36/M	Left index and ring fingers, distal phalanges	Bronchogenic squamous cell carcinoma	none	Amputation of the index finger, chemotherapy, radiotherapy	12
Vine and Cohen [76]	1996	1	57/F	Left thumb, subungual	Renal cell carcinoma	120	Radiotherapy	3
Viswanathan et al. [9]	1996	1	70/M	Right middle finger: distal phalanx + index finger: all the phalanges	Lower alveolus squamous cell carcinoma	24	Radiotherapy, chemotherapy	not reported
Bauer et al. [77]	1997	1	66/F	Right pisiform	Bladder carcinoma	18	not reported	not reported
Hatakeyama et al. [78]	1997	1	72/M	Right first metacarpal	Bronchogenic squamous cell carcinoma	not specified	not reported	not reported
Javed et al. [79]	1997	1	72/F	Right index finger, distal phalanx	Bronchogenic adenocarcinoma	none	not reported	not reported

Lewin et al. [21]	1997	1	70/M	Left thumb, pulp	Laryngeal squamous cell carcinoma	9	not reported	1.5
Mendez Lopez et al. [80]	1997	1	54/M	Right first metacarpal	Colon adenocarcinoma	48	none	1
Rümenapf et al. [81]	1997	1	69/M	Left index finger, distal phalanx	Colon adenocarcinoma	18	Amputation	24
Baron et al. [82]	1998	1	59/F	Left index finger, distal phalanx	Colon adenocarcinoma	120	Radiotherapy	not reported
Delsmann and Caselmann [83]	1998	1	62/M	Right thumb metacarpal and thenar area	Hepatocellular carcinoma	none	Ray amputation, chemotherapy	15
Baran et al. [84]	1998	1	70/F	Left middle finger, distal phalanx	Bronchogenic squamous cell carcinoma	2	not reported	not reported
Celik et al. [85]	1998	1	36/M	Left index and middle fingers, distal phalanges	Bronchogenic mixed squamous and small cell carcinoma	none	Radiotherapy	3
Chin et al. [86]	1998	1	66/F	Right middle finger, PIP joint	Chronic lymphocytic leukemia	48	Chemotherapy	not reported
Chirodian et al. [87]	1998	1	72/M	Left ring finger, distal phalanx	Adenocarcinoma, unknown origin	none	DIP joint disarticulation	1
De Abaffy et al. [19]	1998	1	54/M	Right little finger, proximal phalanx	Bronchogenic large cell carcinoma	4	Transmetacarpal amputation	not reported
Galmarini et al. [88]	1998	1	53/M	Left thumb, distal phalanx	Bronchogenic adenocarcinoma	not specified	Thumb amputation	5
Massraf and Wand [89]	1998	1	62/M	Left thumb, distal phalanx	Prostatic carcinoma	not specified	Amputation through the proximal phalanx	not reported
Umebayashi [90]	1998	1	81/M	Right index finger, distal phalanx	Esophageal squamous cell carcinoma	2	none	1.5
Adegboyega et al. [91]	1999	1	60/M	Right middle finger, middle phalanx	Renal cell carcinoma	none	Amputation of the middle finger	11
Chang et al. [92]	1999	1	57/M	Right thumb, subungual	Bronchogenic squamous cell carcinoma	none	not reported	not reported
Giberti et al. [93]	1999	1	52/M	Right first metacarpal	Renal cell carcinoma	8 mo	Thumb amputation	not reported
Lee et al. [94]	1999	1	47/M	Right thumb, distal phalanx	Hepatocellular carcinoma	10	CMC joint disarticulation	5
Okada et al. [95]	1999	1	84/F	Left ring finger, pulp	Gastric adenocarcinoma	2	Incisional biopsy	12
Vanhooteghem et al. [96]	1999	1	61/M	Right and left little fingers, nails	Bronchogenic squamous cell carcinoma	none	Amputation	5
Yasaka et al. [97]	1999	1	64/M	Left little finger, pulp	Esophageal squamous cell carcinoma	2	not reported	2
Esther and Bos [98]	2000	1	58/F	Right capitate	Parotid mucoepidermoid carcinoma	60	Radiotherapy	not reported
Filloux and Fontaine [99]	2000	6	57/M	Right index finger, pulp	Bronchogenic carcinoma	5	DIP joint disarticulation	1

			76/M	Right carpals, metacarpals, pulp of the index and middle fingers	Renal cell carcinoma	not specified	Upper limb amputation	15
			54/M	Right little finger, pulp	Pharynx cancer	not specified	not reported	1
			58/M	Left middle finger, soft tissue and middle phalanx	Laryngeal squamous cell carcinoma	24	not reported	14
			62/M	Left index finger, pulp	Sigmoid adenocarcinoma	9		15
			72/M	Ring finger, pulp	Bronchogenic carcinoma	none	DIP joint disarticulation	6
Houston and Telepak [100]	2000	1	56/M	Left ring finger, distal phalanx	Esophageal basaloid squamous cell carcinoma	8	Excision	not reported
Ryu et al. [101]	2000	1	63/M	Ring finger, subungual (side not specified)	Bronchogenic squamous cell carcinoma	3	Radiotherapy, chemotherapy	6
Shannon et al. [14]	2000	1	54/M	Right ring finger, distal phalanx	Thymic undifferentiated carcinoma	84	PIP joint disarticulation	2
Tochigi et al. [102]	2000	1	56/F	Left first dorsal interosseous muscle	Palate malignant melanoma	30	Wide excision, chemotherapy	not reported
Asthana et al. [103]	2001	1	40/F	Left thumb, proximal phalanx	Breast carcinoma	not specified	Radiotherapy	6
Caglar and Ceylan [104]	2001	1	51/M	Right capitate	Bronchogenic squamous cell carcinoma	none	not reported	not reported
Chang et al. [105]	2001	1	66/M	Right fourth metacarpal	Gastric adenocarcinoma	none	Radiotherapy	3
Fang et al. [106]	2001	1	49/M	Right hand, soft tissue (multiple cutaneous nodules over the fingers' pulps and palm)	Hepatocellular carcinoma	12	not reported	2
Ghert et al. [32]	2001	1	56/F	Left index finger, middle phalanx	Renal cell carcinoma	12	PIP joint disarticulation	not reported
Henderson and Jehangir [107]	2001	1	52/M	Right ring finger, distal phalanx	Sigmoid colon adenocarcinoma	none	Finger amputation	11
Mehta et al. [108]	2001	1	61/F	Right thumb, subungual	Bronchogenic small cell carcinoma	2	Excision	4
Reichert et al. [28]	2001	1	29/M	Left capitate	Foot clear cell sarcoma	2	Partial excision of the carpus	10
Silfen et al. [109]	2001	1	73/M	Right ring finger, pulp	Esophageal squamous cell carcinoma	none	Chemotherapy	5
Carvalho Hde et al. [110]	2002	1	51/F	Right thumb, distal phalanx	Bronchogenic small cell carcinoma	2	Radiotherapy	12
Parungao and Milner [111]	2002	1	65/M	Right thumb, distal phalanx	Bronchogenic non-small cell carcinoma	none	Thumb amputation	not reported
Raissouni et al. [112]	2002	1	36/F	Right third metacarpal	Bronchogenic adenocarcinoma	none	not reported	0.7

Theunissen et al. [113]	2002	1	63/M	Right thumb and index finger, pulps	Bronchogenic squamous cell carcinoma	none	not reported	not reported
Tolo et al. [10]	2002	1	63/M	Left triquetrum	Renal cell carcinoma	156	En-bloc resection of the ulnar side of the wrist	13
Vadivelu and Drew [114]	2002	1	46/F	Right ring finger, middle phalanx	Breast ductal carcinoma	none	Radiotherapy, chemotherapy	not reported
Aguiar Bujanda et al. [115]	2003	1	65/M	Left thumb, distal phalanx	Esophageal squamous cell carcinoma	3	Pain control with analgesics	not reported
Blanes et al. [116]	2003	1	67/F	Left index finger, distal phalanx	Renal cell carcinoma	not specified	not reported	not reported
Dimri et al. [117]	2003	1	60/M	Right little finger, distal phalanx	Esophageal squamous cell carcinoma	not specified	none	1
Fusetti et al. [118]	2003	1	69/M	Left middle finger, middle phalanx	Renal cell carcinoma	48	PIP joint disarticulation	15
Oron et al. [119]	2003	1	77/F	Left index finger, proximal phalanx	Colon adenocarcinoma	1.5	Ray amputation	not reported
Campa et al. [120]	2004	1	72/F	Right ring finger, distal phalanx	Bronchogenic adenocarcinoma	not specified	DIP joint disarticulation	6
Chou et al. [121]	2004	1	63/M	Right thumb and both ring fingers pulps	Esophageal squamous cell carcinoma	12	not reported	0.5
De Smet [122]	2004	1	71/F	Right second metacarpal	Breast carcinoma	156	Radiotherapy	not reported
Fontana et al. [123]	2004	1	62/M	Right index and middle fingers, middle phalanges	Hepatocellular carcinoma	not specified	Amputation	4
Riter and Ghobrial [124]	2004	1	53/F	Both index fingers, distal phalanges	Renal cell carcinoma	not specified	Right index DIP joint disarticulation	not reported
Sahbaz et al. [125]	2004	1	54/M	Right middle finger, distal phalanx	Bronchogenic squamous cell carcinoma	4	Radiotherapy	not reported
Keramidas and Brotherston [126]	2005	1	66/F	Right middle, ring, little metacarpals, capitates, and hamate	Bronchogenic adenocarcinoma	none	Biopsy	3
Mátrai et al. [20]	2005	1	63/F	Right hand, fourth metacarpal	Colon adenocarcinoma	36	Ray amputation	not reported
Mousavi et al. [127]	2005	1	80/F	Right thumb, distal phalanx	Esophageal papillary adenocarcinoma	not specified	Amputation	not reported
Nakamura et al. [128]	2005	2	73/M	Left thumb, pulp	Bronchogenic squamous cell carcinoma	none	Radiotherapy, chemotherapy	6
			71/M	Left ring finger, pulp	Bronchogenic squamous cell carcinoma	1	not reported	not reported
Ozcanli et al. [23]	2005	3	58/M	Left thumb, distal phalanx	Bladder transitional cell carcinoma	96	Thumb amputation	not reported
			42/F	Right third metacarpal	Colon adenocarcinoma	5	Ray amputation	not reported

			40/M	Right ring finger, subungual	Femur chondrosarcoma	48	Biopsy	not reported
Tomas et al. [129]	2005	1	25/F	Right capitate	Skin malignant melanoma (interscapular)	20	Chemotherapy	not reported
Akjouj et al. [130]	2006	1	57/M	Left thumb, distal phalanx	Bronchogenic adenocarcinoma	none	DIP joint disarticulation	not reported
Bahk et al. [17]	2006	1	67/F	Left hand, all distal phalanges	Gastric adenocarcinoma	42	Shoulder disarticulation	4
Bülbül et al. [131]	2006	1	67/M	Right little finger, middle phalanx	Bronchogenic non-small cell carcinoma	not specified	Chemotherapy	not reported
Carty et al. [132]	2006	1	68/F	Left middle finger, proximal phalanx	breast carcinoma	48	Ray amputation	not reported
Gallagher et al. [133]	2006	1	72/M	Left thumb, subungual	Rectal adenocarcinoma	60	MCP joint disarticulation	6
Gamblin et al. [134]	2006	1	72/M	Index finger, distal phalanx	Sigmoid colon adenocarcinoma	not specified	Ray amputation	not reported
Gawley et al. [135]	2006	1	65/M	Left little finger, distal phalanx	Bronchogenic squamous cell carcinoma	none	Digital amputation	3
Heidarpour et al. [136]	2006	1	78/F	Left index finger, distal phalanx	Bronchogenic carcinoma	none	none	2
Park et al. [137]	2006	1	39/F	Right trapezium, left capitates	Gastric adenocarcinoma	not specified	Radiotherapy, chemotherapy	not reported
Reparaz Padros et al. [138]	2006	1	61/F	Left ring finger, distal phalanx	Thyroid follicular carcinoma	84	Amputation through the middle phalanx	not reported
Afshar et al. [139]	2007	1	33/F	Right little finger, pulp	Uterine choriocarcinoma	not specified	DIP joint disarticulation	11
Bigot et al. [140]	2007	1	64/M	Right third metacarpal	Gastric adenocarcinoma	12	Excision	5
Elhassan and Fakhouri [141]	2007	1	68/M	Left first metacarpal	Bronchogenic squamous cell carcinoma	none	Wide amputation	not reported
French et al. [142]	2007	1	39/F	Right hand, extensive cutaneous necrosis	Breast ductal adenocarcinoma	12	Right shoulder disarticulation	18
Kanbay et al. [143]	2007	1	47/M	Left index finger, soft tissue nodule	Pleural malignant mesothelioma	not specified	none	6
Nikolic et al. [144]	2007	1	67/M	Right thumb, proximal phalanx	Colorectal adenocarcinoma	30	Transmetacarpal amputation	39
Preto et al. [145]	2007	1	61/M	Right trapezoid, base of the first and second metacarpals	Bronchogenic adenocarcinoma	none	not reported	6
Ramseier et al. [146]	2007	1	76/M	Right thumb and left ring finger, subunguals	Foot chondrosarcoma	60	marginal excision (ring finger), en-bloc resection (thumb)	not reported
Salesi et al. [147]	2007	1	74/M	Left index finger, proximal phalanx and right first metacarpal	Renal cell carcinoma	36	Amputation of the	not reported
Ahlmann et al. [148]	2008	3	65/M	Right hamate	Bronchogenic adenocarcinoma	none	Excision of the hamate	12

			73/M	Left ring finger, proximal phalanx	Bronchogenic non-small cell carcinoma	none	Ray amputation	3
			62/F	Left thumb, distal and proximal phalanges	Bronchogenic adenocarcinoma	12	MCP joint disarticulation	not reported
Chen et al. [149]	2008	1	41/F	Left middle finger, pulp	Mediastinal epitheloid angiosarcoma	not specified	Chemotherapy, radiotherapy	2
Flynn et al. [6]	2008	2	78/F	Left second metacarpal	Bronchogenic non-small cell carcinoma	none	Radiotherapy	not reported
			65/F	Right third and fifth metacarpals	Breast carcinoma	not specified	Radiotherapy	not reported
Gaston et al. [150]	2008	1	45/F	Right trapezium	Bronchogenic non-small cell carcinoma	not specified	Excision of the trapezium	not reported
Hsieh et al. [24]	2008	1	56/M	Left thumb (bone not specified)	Esophageal squamous cell carcinoma	3	Chemotherapy	not reported
Kanatani et al. [151]	2008	1	57/M	Left little finger, distal phalanx	Esophageal carcinoma	24	PIP joint disarticulation	2
Miyamoto et al. [152]	2008	1	72/F	Left fifth metacarpal	Gastric adenocarcinoma	24	Wide excision	12
Ottomani et al. [153]	2008	1	52/M	Left index and right middle fingers' pulps	Laryngeal squamous cell carcinoma	9	Amputation of left, excision of right middle finger pulp	not reported
Plotkine et al. [154]	2008	1	80/M	Right middle finger, distal phalanx	Nasopharynx carcinoma	60	Amputation through the proximal phalanx	not reported
Seth et al. [155]	2008	1	46/M	Right ring finger, subungual	Bronchogenic adenocarcinoma	none	Marginal excision	4
Spiteri et al. [4]	2008	1	82/M	Right ring finger, distal phalanx	Gastric adenocarcinoma	none	Ray amputation	not reported
Tzaveas et al. [156]	2008	1	68/M	Right fifth metacarpal	Bronchogenic carcinoma	5	Chemotherapy	not reported
Jakhar et al. [157]	2009	1	70/M	Right ring finger, proximal phalanx	Bronchogenic adenocarcinoma	none	Radiotherapy, chemotherapy	not reported
Kodama et al. [158]	2009	1	78/M	Left hamate	Bronchogenic adenocarcinoma	2	Radiotherapy, chemotherapy	22
Majidi et al. [159]	2009	1	55/M	Right index finger, distal phalanx	Bronchogenic squamous cell carcinoma	none	DIP joint disarticulation	2
Mitrovic et al. [160]	2009	1	67/M	Left ring finger, proximal phalanx	Renal cell carcinoma	none	Ring finger amputation	not reported
Otsuji et al. [161]	2009	1	49/F	Left little finger distal phalanx	Hepatocellular carcinoma	60	CMC joint disarticulation	not reported
Rauh et al. [162]	2009	1	69/M	Right little finger, distal phalanx	Skin squamous cell carcinoma (right thumb, distal phalanx)	not specified	Ray amputation	6
Shrivastava et al. [163]	2009	1	66/M	Left middle finger, middle phalanx	Lower alveolus squamous cell carcinoma	12	Radiotherapy, chemotherapy	3

Wavreille et al. [164]	2009	1	48/F	Right little finger, distal phalanx	Breast carcinoma	108	DIP joint disarticulation	not reported
Afshar and Ilkhanizadeh [165]	2010	1	72/M	Right thumb, pulp	Leukemia cutis, chronic lymphocytic leukemia	not specified	IP joint disarticulation	not reported
Anoop et al. [166]	2010	1	76/M	Right little finger, distal phalanx	Colon well-differentiated adenocarcinoma	none	Radiotherapy, chemotherapy	1
Anandan et al. [167]	2010	1	64/M	Left middle finger, proximal phalanx	Bronchogenic small cell carcinoma	6	Radiotherapy	3
Anglada-Curado et al. [168]	2010	1	75/F	Left hypothenar (soft tissue)	Renal clear cell carcinoma	12	Marginal excision	9
Biyi et al. [169]	2010	1	37/F	Left thumb, proximal phalanx	Breast adenocarcinoma	48	Chemotherapy	18
Borobio León et al. [170]	2010	1	81/F	Left third metacarpal	Rectal adenocarcinoma	12	Radiotherapy, chemotherapy	7
Chakravarthy et al. [171]	2010	1	32/F	Right hand, metacarpal	Thyroid papillary carcinoma	not specified		not reported
Long et al. [172]	2010	1	53/M	Left middle finger, distal phalanx	Bronchogenic adenosquamous cell carcinoma	none	none	1
Lozic et al. [173]	2010	1	68/M	Right thumb, distal phalanx	Bronchogenic squamous cell carcinoma	none	Chemotherapy	4
Lucilli et al. [174]	2010	1	63/M	Left thumb distal phalanx	Bronchogenic squamous cell carcinoma	24	IP joint disarticulation	6
Myrehaug and Bezjak [175]	2010	1	age not specified/M	Right index finger, proximal phalanx	Bronchogenic adenocarcinoma	not specified	Radiotherapy	not reported
Vasic [176]	2010	1	52/M	Right thumb, proximal and distal phalanges	Sigmoid colon	not specified	Radiotherapy	not reported
Wurapa et al. [177]	2010	1	80/F	Left scaphoid, trapezium and trapezoid	Esophageal adenocarcinoma	36	Below-elbow amputation	7
Amar et al. [178]	2011	1	46/F	Left thumb, distal phalanx	Bronchogenic adenocarcinoma	none	MCP joint disarticulation	11
Bhandari and Brown [27]	2011	1	52/M	Left index finger, proximal phalanx	Bronchogenic adenocarcinoma	none	Ray resection	not reported
Bowles and Wells [179]	2011	1	63/M	Right ring finger, distal phalanx	Bronchogenic carcinoma	none	not reported	not reported
Çetın et al. [180]	2011	1	53/M	Right thenar muscles eminence	Rectal adenocarcinoma	24	Partial resection	not reported
Chao et al. [181]	2011	1	62/F	Left middle finger, proximal phalanx	Bronchogenic squamous cell carcinoma	not specified	Radiotherapy	not reported
Dar et al. [16]	2011	1	75/M	Both hands, distal phalanges of all the digits	Esophageal squamous cell carcinoma	36	Amputation of the fifth finger, chemotherapy	not reported
Elvey et al. [182]	2011	1	67/M	Left second, third, fourth and fifth metacarpals	Parotid basal cell adenocarcinoma	24	Below elbow amputation	not reported
Huri [183]	2011	1	84/M	Right thumb, distal phalanx	Thyroid follicular-type adenocarcinoma	not specified	Amputation through the proximal phalanx	not reported

Jenzer et al. [184]	2011	1	85/M	Right ring finger, proximal phalanx	Gastroesophageal adenocarcinoma	not specified	Ray amputation	2
Kontogeorgakos et al. [185]	2011	1	75/F	Right middle finger, distal phalanx	Bronchogenic non-small cell carcinoma	72	Ray amputation	15
Kumar et al. [186]	2011	3	60/M	Right thumb, distal phalanx, onychlysis	Skin squamous cell carcinoma (right inguinal)	96	Radiotherapy	not reported
			55/M	Left hand, all 5 distal phalanges	Laryngeal supraglotic squamous cell carcinoma	24	Supportive	not reported
			52/M	Left little finger, distal phalanx	Esophageal squamous cell carcinoma	none	Radiotherapy	not reported
Roohi et al. [187]	2011	1	57/F	Most bones of the right hand except scaphoid, trapezium, thumb phalanges, and second metacarpal	Rectal adenocarcinoma	60	Below-elbow amputation	not reported
Sonoda et al. [25]	2011	1	70/F	Right index finger, middle phalanx	Renal cell carcinoma	not specified	not reported	not reported
Taleb et al. [188]	2011	1	46/F	Left fourth metacarpal	Bladder urothelial carcinoma	24	Radiotherapy, excision of the metacarpal	not reported
Verardino et al. [189]	2011	1	59/F	Right and left ring fingers, distal phalanges	Anal canal basaloid carcinoma	36	Radiotherapy	not reported
Borgohain et al. [190]	2012	1	70/M	Right second metacarpal, trapezium and trapezoid	Renal cell carcinoma	none	none	not reported
Corrales Pinzón et al. [191]	2012	1	59/M	Right wrist, all carpals	Hepatocellular carcinoma	none	Splint, pain control	not reported
Fadli et al. [192]	2012	1	67/F	Left first metacarpal	Rectosigmoid adenocarcinoma	24	not reported	not reported
Ferraro and Lucero [193]	2012	1	52/F	Left thumb, proximal phalanx	Bronchogenic adenocarcinoma	none	not reported	not reported
Gharwan et al. [194]	2012	1	54/M	Right third metacarpal	Colon adenocarcinoma	none	Ray amputation	not reported
Kamolz et al. [195]	2012	1	69/M	Left palm	Esophagogastric junction adenocarcinoma	12	Chemotherapy	not reported
Khosla et al. [29]	2012	1	65/F	Left fourth metacarpal	Vaginal squamous cell carcinoma	11	Radiotherapy, chemotherapy	not reported
Kim et al. [196]	2012	1	55/M	Left thumb, skin of the distal phalanx	Hepatocellular carcinoma	36	PIP joint disarticulation	6
Ornetti et al. [197]	2012	1	68/F	Right index finger, soft tissue	Uterine endometrial adenocarcinoma	not specified	Treatment refused	6
Ragois et al. [198]	2012	1	72/M	Left hypothenar (soft tissue)	Bronchogenic small cell carcinoma	48	Excision, skin graft	2
Rauf et al. [199]	2012	1	66/M	Left little finger, distal phalanx	Hepatocellular carcinoma	none	Excision	not reported

Rinonapoli et al. [200]	2012	1	74/M	Left trapezium, trapezoid and scaphoid	Bronchogenic undifferentiated large cell carcinoma	none	Below-elbow amputation	not reported
Roushdi et al. [201]	2012	1	66/F	Left hamate	B-cell lymphoma	none	Chemotherapy	not reported
Sipahioglu et al. [18]	2012	1	60/M	Middle finger, distal phalanx, pulp (side not specified)	Nasopharynx epidermoid carcinoma	36	MCP joint disarticulation	24
Song and Yao [30]	2012	1	70/M	Right trapezium	Bronchogenic non-small cell carcinoma	none	Trapeziumectomy	not reported
Stahl et al. [202]	2012	1	46/F	Left scaphoid	Lower leg melanoma	96	Excision	13
Tan et al. [203]	2012	1	71/M	Right fourth metacarpal	Renal cell carcinoma	none	Radiotherapy, chemotherapy	13
Khmamouche et al. [204]	2013	1	72/M	Left thumb, distal phalanx	Bronchogenic adenocarcinoma	none	Thumb amputation	1
Krishnamurthy and Ramshankar [205]	2013	1	45/M	Left third metacarpal	Thyroid follicular carcinoma	none	Idodine-131 ablation + radiotherapy	not reported
Yoneda et al. [206]	2013	1	64/M	Left ring finger, distal phalanx	Bladder cancer	not specified	Finger amputation	3
Gilardi et al. [207]	2013	1	55/M	Left trapezium-metacarpal joint	Bronchogenic adenocarcinoma	none	Radiotherapy, chemotherapy	4
Rommer et al. [208]	2013	2	30/M	Left ring finger, second metacarpal, fourth distal phalanx	Hepatocellular carcinoma	none	middle phalanx of the ring and small fingers amputation	not reported
			66/F	Right index finger, left middle finger	Renal clear cell carcinoma	not specified	distal phalanx of the right index finger and middle phalanx of the left middle finger amputation	4
van Veenendaal et al. [209]	2014	1	83/F	Right middle finger	Bronchogenic adenocarcinoma	not specified	right middle finger amputation	not reported
Hernández-Cortés et al. [210]	2014	1	53/M	Right fifth finger	Renal clear cell carcinoma	5	Proximal phalanx amputation	3
Sumodhee et al. [211]	2014	1	61/M	Left ring finger	Bronchogenic adenocarcinoma	not specified	Radiotherapy, chemotherapy	12
Liu et al. [212]	2014	1	53/F	Left index finger	Bronchogenic adenocarcinoma	4	Excisional biopsy, radiotherapy, chemotherapy	12
Lambe et al. [213]	2014	1	72/M	Right fifth finger, distal phalanx	Bronchogenic squamous cell carcinoma	not specified	Radiotherapy	0.5
Gorospe Sarasúa et al. [214]	2015	1	43/F	Left ring finger, middle phalanx	Bronchogenic adenocarcinoma	none	not reported	not reported
Reynolds and Skandan [215]	2016	1	71/F	Left index finger, distal phalanx	Bronchogenic adenocarcinoma	none	distal phalanx of the left index finger amputation	2
Sahoo et al. [216]	2016	1	79/M	Left index finger, distal phalanx	Bronchogenic squamous cell carcinoma	none	distal phalanx of the left index finger amputation	not reported
Asirvatham Gjorup et al. [217]	2016	1	55/F	Right middle finger, middle phalanx	Bronchogenic non-small cell carcinoma	none	Excision	2

Muñoz-Mahamud et al. [218]	2016	5	63/M	First metacarpal	Bronchogenic adenocarcinoma	none	none	6
			52/F	Left middle finger, middle phalanx	Bronchogenic squamous cell carcinoma	none	Transmetacarpal amputation	12
			25/F	right capitate	melanoma (interscapular region)	20	Biopsy	1
			36/F	Right ring finger, distal phalanx	squamous cell carcinoma of the cervix	not specified	Partial excision	4
			54/M	Left thumb, distal phalanx	Renal clear cell carcinoma	not specified	none	2
Xie and Huang [219]	2017	1	45/M	Left thumb, proximal phalanx	Bronchogenic adenocarcinoma	36	Thumb amputation	not reported
Lechmiannandan et al. [220]	2017	1	52/M	Right thumb, distal phalanx	Renal clear cell carcinoma	7	Thumb amputation	6
El Idrissi et al. [221]	2018	1	68/M	Fifth metacarpal	Bronchogenic adenocarcinoma	3	Amputation	not reported
Tabrizi et al. [222]	2018	1	60/M	Left hamate	Bronchogenic adenocarcinoma	none	Biopsy	20
Voskuil et al. [223]	2019	1	81/M	Right scaphoid	Adenocarcinoma, unknown origin	none	Proximal row carpectomy	12
Ho et al. [224]	2020	1	55/F	Right ring finger, distal phalanx	Renal clear cell carcinoma (Von Hippel-Lindau syndrome)	none	Biopsy	not reported
Hirano et al. [225]	2020	1	70/M	Right middle finger, distal phalanx	Bronchogenic squamous cell carcinoma	none	not reported	not reported
Cruz et al. [226]	2020	1	62/M	Second, third, fourth metacarpals	Chronic lymphocytic leukemia	48	Biopsy	not reported
Gallardo-Alvarado et al. [227]	2020	1	58/F	Left thumb, distal phalanx	squamous cell carcinoma of the cervix	6	none	4

Supplementary Table 1: Patient's demographics.
